# A159 SERUM IGG RESPONSE TO THE D0-D1 HINGE PEPTIDE OF COMMENSAL FLAGELLIN PREDICTS THE FUTURE ONSET OF CROHN’S DISEASE IN HEALTHY FIRST-DEGREE RELATIVES

**DOI:** 10.1093/jcag/gwae059.159

**Published:** 2025-02-10

**Authors:** R Y Wu, M Xue, Q Zhao, S Jeong, A Griffiths, L Dieleman, A Steinhart, G Aumais, B Bressler, R Panaccione, C Deslandres, D Mack, C N Bernstein, J Marshall, D Turner, W Xu, L W Duck, C Elson, W Turpin, S Lee, K Croitoru

**Affiliations:** Division of Gastroenterology and Hepatology, Temerty Faculty of Medicine, University of Toronto, Toronto, ON, Canada; Inflammatory Bowel Disease Centre, Division of Gastroenterology, Mount Sinai Hospital, Toronto, ON, Canada; Department of Medicine, University of Alabama at Birmingham, Birmingham, AL; Inflammatory Bowel Disease Centre, Division of Gastroenterology, Mount Sinai Hospital, Toronto, ON, Canada; Division of Gastroenterology, The Hospital for Sick Children, Toronto, ON, Canada; Division of Gastroenterology, Department of Medicine, University of Alberta, Edmonton, AB, Canada; Inflammatory Bowel Disease Centre, Division of Gastroenterology, Mount Sinai Hospital, Toronto, ON, Canada; Hôpital Maisonneuve-Rosemont, Department of Medicine, University of Montreal, Montreal, QC, Canada; The Inflammatory Bowel Disease Centre of British Columbia, University of British Columbia, Vancouver, BC, Canada; Inflammatory Bowel Disease Clinic, Division of Gastroenterology and Hepatology, University of Calgary, Calgary, AB, Canada; Department of Hepatology and Pediatric Nutrition, Centre Hospitalier Universitaire Sainte-Justine, Montreal, QC, Canada; Division of Gastroenterology, Hepatology & Nutrition, Children’s Hospital of Eastern Ontario and University of Ottawa, Ottawa, ON, Canada; University of Manitoba Inflammatory Bowel Disease Clinical and Research Centre and, Department of Internal Medicine, Max Rady College of Medicine, Rady Faculty of Health Sciences, University of Manitoba, Winnipeg, MB, Canada; Department of Medicine, Farncombe Family Digestive Health Research Institute, McMaster University, Hamilton, ON, Canada; The Juliet Keidan Institute of Pediatric Gastroenterology and Nutrition, Shaare Zedek Medical Center, The Hebrew University of Jerusalem, Jerusalem, Israel; Division of Biostatistics, Dalla Lana School of Public Health, University of Toronto, Toronto, ON, Canada; Department of Medicine, University of Alabama at Birmingham, Birmingham, AL; Department of Medicine, University of Alabama at Birmingham, Birmingham, AL; Inflammatory Bowel Disease Centre, Division of Gastroenterology, Mount Sinai Hospital, Toronto, ON, Canada; Inflammatory Bowel Disease Centre, Division of Gastroenterology, Mount Sinai Hospital, Toronto, ON, Canada; Inflammatory Bowel Disease Centre, Division of Gastroenterology, Mount Sinai Hospital, Toronto, ON, Canada

## Abstract

**Background:**

Elevated antimicrobial antibody levels have been reported up to 6 years before diagnosis of Crohn’s disease (CD), but the specific antibody response is poorly defined.

**Aims:**

Here, we characterized the nature of the antimicrobial antibody responses before the diagnosis of CD in healthy first-degree relatives (FDR) of CD patients.

**Methods:**

The CCC-GEM Project nested case-control cohort consisted of FDRs who later developed CD (n=77), matched 1:4 by age, sex, follow-up duration, and geographical location with FDRs who remained healthy (n=304). Sera at enrollment were probed for antimicrobial reactivity using a microbiota antigen microarray and a flagellin peptide cytometric bead array. Conditional logistic regression was used to assess association with CD onset and partial Spearman was used to correlate the serologic responses with gut permeability (fractional urinary excretion of lactulose-to-mannitol ratio (LMR)) and fecal calprotectin (FCP).

**Results:**

19 (out of 49) IgG antimicrobial antibody responses were significantly associated with the risk of CD; these antibodies were reactive to *Lachnospiraceae* family, particularly *Roseburia*-derived flagellins (*q*<0.02); 5 antibodies positively correlated with FCP (*q*<0.046), whereas 3 antibodies positively correlated with LMR (*q*<0.049). These IgG-seroreactive flagellins shared significant amino acid sequence homology, characterized by a conserved “hinge peptide” within D0-D1 domains of the amino-terminus. Elevated IgG seroreactivity to this hinge peptide (determined by cytometric bead array) was associated with multi-flagellin reactivity and future risk of CD.

**Conclusions:**

Increased antibody response in healthy asymptomatic FDRs towards *Lachnospiraceae* flagellins is associated with future risk of CD, and markers of gut inflammation and altered intestinal permeability. Importantly, pre-CD participants shared seroreactivity towards a conserved bacterial flagellin epitope, suggesting a possible role in the early immunopathogenesis of CD.

**Funding Agencies:**

CCC, CIHRThe Leona M. and Harry B. Helmsley Charitable Trust

